# Structural and functional changes in maternal left ventricle during pregnancy: a three-dimensional speckle-tracking echocardiography study

**DOI:** 10.1186/1476-7120-13-6

**Published:** 2015-01-27

**Authors:** Juan Cong, Tingpan Fan, Xiaoqian Yang, Jared Wynn Squires, Guomei Cheng, Linlin Zhang, Zhan Zhang

**Affiliations:** Department of Ultrasonic Diagnosis, The Third Affiliated Hospital of Zhengzhou University, Zhengzhou, 450052 Henan Province China; Department of Obstetrics and Gynecology, The Third Affiliated Hospital of Zhengzhou University, Zhengzhou, 450052 Henan Province China; Henan Provincial Engineering Research Center for Perinatal Medicine, The Third Affiliated Hospital of Zhengzhou University, Zhengzhou, 450052 Henan Province China; Henan Translational Medicine Engineering Laboratory for Maternal and Children’s Health, The Third Affiliated Hospital of Zhengzhou University, Zhengzhou, 450052 Henan Province China; Department of Surgical Oncology, Massachusetts General Hospital, Boston, 02114 MA USA

**Keywords:** Pregnancy, Remodeling, Myocardial deformation, Three-dimension, Speckle-tracking echocardiography

## Abstract

**Background:**

Pregnancy represents a physiological adaptation to the transient load changes of maternal heart. This study aimed to investigate maternal left ventricle (LV) performance during normal pregnancy by three-dimensional speckle-tracking echocardiography (3D STE) parameters considering LV loading and shape.

**Methods:**

Sequential two-dimensional echocardiography (2DE) and 3D STE were performed on 68 women during each pregnancy trimester and 6 to 9 weeks after delivery, while thirty age-matched, healthy, nonpregnant women served as controls. Global longitudinal strain (GLS), global circumferential strain (GCS), global area strain (GAS) and global radial strain (GRS) were measured.

**Results:**

Increased cardiac index and progressive eccentric hypertrophy was detected, which subsequently recovered postpartum. In late pregnancy, GLS, GCS, GAS and GRS significantly decreased (P < 0.05) accompanied by a slight reduction of LV ejection fraction (EF) (P < 0.05), and these values returned postpartum to baseline level. All 3D strain indices correlated well with gestation age (P < 0.01), while compared to other components, GAS exhibited the strongest association with 3D EF (r = 0.549) and sphericity index (r = 0.328), and was the only parameter that correlated well with LV mass index (r = 0.22).

**Conclusions:**

This study gives normal ranges of 3D STE indices in pregnancy. 3D STE demonstrated modified myocardial deformation and changes in maternal LV structure and function during the gestation period.

## Background

During pregnancy, there are important hemodynamic variations which result in a physiological situation of transient changes preload and afterload in the maternal heart. Those changes are necessary for the progression of a successful pregnancy, but which may also impose further load on the heart. Moreover, heart disease is the leading cause of nonobstetric mortality during pregnancy [1], and the number of pregnant women at risk for cardiovascular complications is on the rise [2]. Therefore, identification and understanding maternal cardiac structure and function is of clinical importance and is essential for the management of cardiology patients in pregnancy.

Despite many reports on maternal cardiac adaption being published, there is controversy about the change in left ventricular (LV) performance during pregnancy. Although it is reported that an increased cardiac output (CO) is paralleled by a decreased peripheral vascular resistance, the documents on the enlargement of chamber size, LV wall thickness and mass are inconsistent [3, 4]. More controversial is in regards to the adjustment of LV functions. The parameters of myocardial systolic function, including ejection fraction (EF), shortening fraction and tissue Doppler velocity, have been variously described as decreased [5], increased [6] or remained constant [7]. These parameters are load dependent, so the use of them is limited by the variations in ventricular loading conditions in pregnancy.

Recently, two-dimensional echocardiographic (2DE) indices of myocardial longitudinal deformation, strain imaging, have been shown decreased significantly in late pregnancy while the traditional parameters did not reflect those functional changes [8]. However, the accuracy of 2D strain could be affected by the use of foreshortened views and out-of-plane motion [9]. Further technological advance of three-dimensional (3D) speckle-tracking echocardiography (STE) has developed to obtain all components of myocardial displacement vectors, which could detect 3D cardiac motion that has been ignored in current 2D STE [10]. 3D STE is more representative of the morphological state of the heart and provides a new opportunity to further our understanding myocardial deformation [11]. In the study, we assessed the time course of changes in LV geometry and functions during normal pregnancy, as well as the effects of normal pregnancy on LV mechanics by 3D STE parameters while considering LV loading and shape.

## Methods

### Study subjects

All women with singleton pregnancy were recruited consecutively as cases, and thirty age-matched, healthy, nonpregnant women served as controls after informed consent and with approval from the Third Affiliated Hospital of Zhengzhou University Ethics Committee. Enrolled criteria of healthy pregnant women was that they were without medical diseases, such as cardiovascular disorders, renal disease etc., and without obstetrical complications, such as gestational diabetes mellitus or pregnancy-induced hypertension. Subjects who had poor echo quality or any fetal abnormalities were excluded from the study. A total of 68 patients were entered into the study. Four visits were planned during the study: trimester 1, 12–14 weeks; trimester 2, 24–26 weeks; trimester 3, 36–38 weeks and 6–9 weeks after delivery. The pregnancy trimester was confirmed by ultrasound scanning in the first half of pregnancy. At each visit, the normal course of pregnancy was confirmed by an obstetric assessment, weight and blood pressure were measured, and an echocardiographic examination was performed.

### 2D echocardiographic examination

Standard 2D echocardiographic examinations, including parasternal and apical views with pulsed Doppler evaluation, were performed with subjects in the left lateral decubitus position using a commercially available ultrasound machine and transducer (M5S transducer, Vivid E9; GE Healthcare, Horten, Norway). The long axis views of the left ventricle were obtained at the apical four-chamber, two-chamber, and long-axis planes, while three short-axis views were acquired at the basal, midventricular and apical level. The following parameters were performed by M-Mode in the parasternal long-axis view as recommended: interventricular septum (IVSd), posterior wall (PWd), left ventricular end-diastolic (LVEDd) and end-systolic (LVEDs) diameters. LVEF and stroke volume (SV) were calculated as previously described [12]. Relative wall thickness (RWT) was calculated as (IVSd + PWd)/LVEDd. Cardiac indices were normalized for body surface area. All image acquisitions were performed at three consecutive beats during breath-holds.

### 3D echocardiographic examination

A full-volume scan was acquired by a matrix-array transducer (V4 transducer, Vivid E9; GE Healthcare, Horten, Norway). Consecutive three-beat electrocardiographically gated subvolume acquisition was performed from the apical approach during apnea to generate the full-volume data set. Frame rate (in volume per second) higher than 40% of the individual heart beat was used in order to increase the possibility that the ‘speckles’ could be recognizable in successive frames. A12-slice display mode available on the machine was selected to ensure the entire LV cavity and walls were included in the full volume. When the acquisition was considered suboptimal, the data set was re-acquired. All data sets were stored digitally in a raw-data format and exported to separate workstations equipped with commercially available quantitative software.

**Figure 1:**
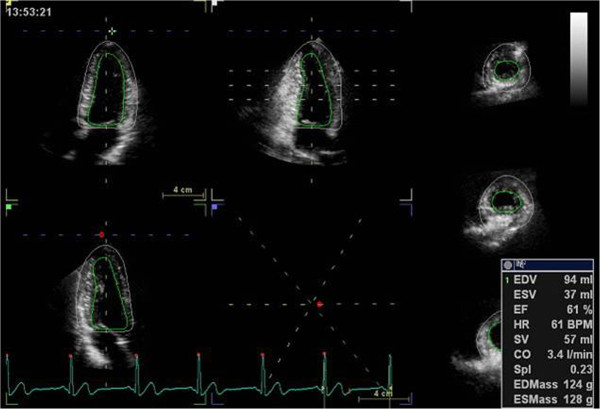
**Automated detection of the endocardium and epicardial border throughout the whole cardiac cycle.** Consequently end-diastolic volume (EDV), end-systolic volume (ESV), ejection fraction (EF), stroke volume (SV), cardiac output (CO), sphericity index(Spl) and LV mass either at end-diastole (EDMass) or at end-systole (ESMass) were automatically calculated.

**Figure 2:**
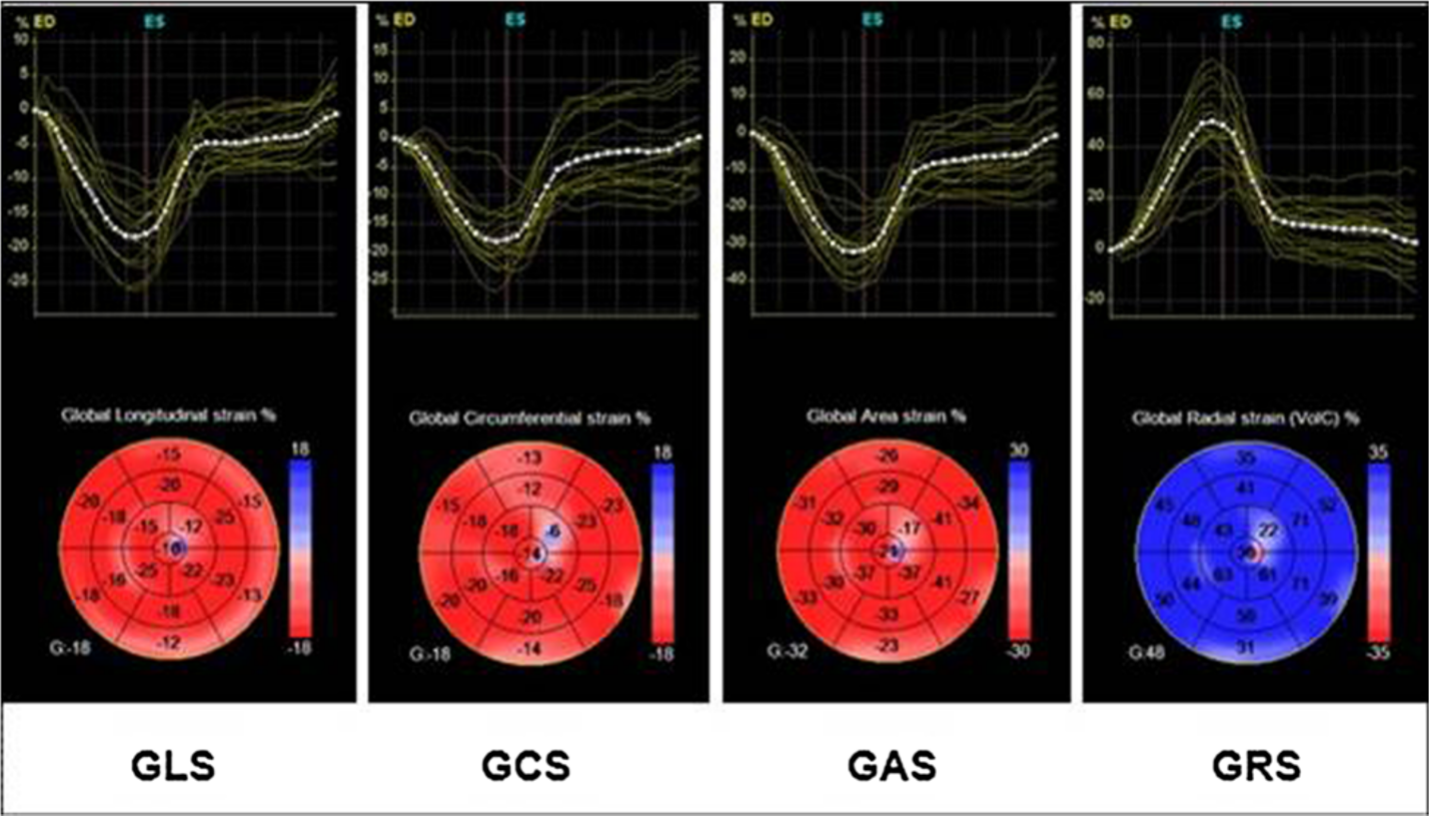
**Both strain curves and a color-coded 17-segment bull’s eye plot were presented.** Color lines indicate regional strain; white dotted line means global (average) strain. Values of longitudinal strain, circumferential strain, and area strain are negative (sign -), whereas values of radial strain are positive (sign +). GAS indicates global area strain; GCS, global circumferential strain; GLS, global longitudinal strain; GRS, global radial strain.

Three-dimensional full-volume data sets were analyzed using the 4DAutoLVQ package (EchoPAC PC version 110.1.8). A quad view, which displayed at end-diastolic frame, was used for manual alignment of the axis and the mitral valve leaflet. Then, in each of the three apical views, three points were indicated manually, including both sides of mitral annular corners and one apical point. Automated border detection followed the endocardium throughout the whole cardiac cycle; subsequently, a frame-by-frame point-and-click correction was manually performed by the operator to adjust the tracking of the endocardium for all segments. After that, end-diastolic volume (EDV), end-systolic volume (ESV), SV, CO, EF and sphericity index (SpI) were automatically calculated (Figure 1). Next, to calculate LV mass and myocardial strain, the epicardial border was determined for manual adjustment of the region of interest. After 3D speckle-tracking were utilized in frame-by-frame analysis, the values of regional and global directional strains (longitudinal, circumferential, and radial) as well as the area strain were generated and presented as strain curves and a color-coded 17-segment bull’s eye plot (Figure 2). Global longitudinal strain (GLS), global circumferential strain (GCS), global area strain (GAS), and global radial strain (GRS) were calculated as the methods reported previously in detail [13]. Using the software, if more than three segments were rejected, global strain values were not calculated. In the present study, patients with ≥3 rejected segments were excluded from statistical analysis.

### Intraobserver and interobserver agreement

All imaging data was analyzed by one observer in random order. To test intraobserver variability, a single observer analyzed the data twice on occasions separated by an interval of 1 month. To test interobserver variability, a second observer analyzed the data without knowledge of the measurements of the first observer.

### Statistical analyses

Descriptive data are shown as means ± SD. ANOVA for repeated measures was used to compare data between sequential studies in the pregnant group. Pregnancy data were compared with controls using independent sample *t* tests. 3D strain data were compared with 2D approach by paired *t* test. Pearson correlation coefficient was used to analyze the relationship between two parameters. Reproducibility was assessed as the mean percentage error (absolute difference divided by the mean of the 2 observations). We used SPSS version 17.0 (SPSS, Inc) statistical software. *P* < 0.05 was considered to indicate statistical significance.

## Results

### Study subjects

From the initially enrolled 87 participants, those with poor-quality images (5 participants), lacking at least 2 visits during pregnancy (6 participants), and those pregnancy-related pathology (1 late miscarriage, 2 gestational diabetes mellitus, 1 mild preeclampsia and 2 arterial hypertension during pregnancy) and with fetal abnormalities (2 participants) were excluded from the analysis. Finally, sixty-eight pregnant women were involved in the study. Their mean age was 29.6 ± 4.2 years (range 26 ~ 33 years). The mean age of the 30 participants of the control group was 30 ± 5.1 years (*P* not significant).

### Evolution of clinical and hemodynamic characteristics

Table 1 summarizes the clinical and hemodynamic characteristics of the study population. Diastolic blood pressure and mean blood pressure were slightly reduced during the second trimester but followed by mild increase toward the third trimester. The cardiac index increased progressively by a mean of 33% between the first and third trimesters principally as a result of late increase in heart rate and in stroke volume.

**Table 1:** **Clinical and hemodynamic data**

Variable	Controls	Trimester 1	Trimester 2	Trimester 3	Postpartum
	-	(14.1 ± 1.6)wk	(25.5 ± 2.6)wk	(37.8 ± 2.4)wk	(7.8 ± 2.4)mo
Weight(kg)	58.3 ± 11.4	60.1 ± 9.2	68.2 ± 9.5*^†‡§^	72.8 ± 7.0*^†^	61.8.3 ± 13.4^‡^
BSA(m^2^)	1.62 ± 0.13	1.64 ± 0.20	1.66 ± 0.11‡	1.74 ± 0.10*^†^	1.65 ± 0.15^‡^
Heart rate(bpm)	81.1 ± 14.3	82.6 ± 10.4	85.1 ± 17.2	90.1 ± 9.8*^†^	80.3 ± 14.2^‡^
SBP(mmHg)	104.2 ± 10.7	103.0 ± 6.7	102.9 ± 8.5	107.6 ± 9.6	110.2 ± 10.3
DBP(mmHg)	64.4 ± 8.0	64.3 ± 4.9	61.3 ± 6.8^‡§^	67.2 ± 7.9	68.6 ± 6.0
MBP(mmHg)	79.3 ± 8.5	79.5 ± 5.0	76.5 ± 6.2^‡§^	81.7 ± 7.7	83.3 ± 8.1
CI (l · min-1 · m-2)	2.98 ± 0.76	3.01 ± 0.52	3.59 ± 0.79*	4.06 ± 0.72*^†^	3.36 ± 0.72^‡^
SVI(ml/m^2^)	34.58 ± 6.23	34.69 ± 9.60	37.56 ± 8.52	39.03 ± 5.34*^†^	36.56 ± 7.54

Data are given as mean ± SD; BSA indicates body surface area; SBP, systolic blood pressure; DBP, diastolic blood pressure; MBP, mean blood pressure; CI, cardiac index; SVI, stroke volume index.

*P < 0.05 vs. Controls; ^†^ *P* < 0.05 vs. Trimester 1; ^‡^ *P* < 0.05 vs. Trimester 3; ^§^ *P* < 0.05 vs. Postpartum.

### Evolution of 2D echocardiographic assessment

Data of 2DE parameters are listed in Table 2. A progressive increase in LV diameters and LV wall thickness, consistent with the development of slight eccentric hypertrophy, was detected during pregnancy, which subsequently recovered postpartum. Of note, during the first trimester, indices of LV systolic function in pregnant women were similar to controls, however, both EF and s decreased slightly between the second and third trimesters, which turned to the level of control group after delivery.

**Table 2:** **Two-dimensional echocardiographic parameters and myocardial velocities**

Variable	Controls	Trimester 1	Trimester 2	Trimester 3	Postpartum
	-	(14.1 ± 1.6)wk	(25.5 ± 2.6)wk	(37.8 ± 2.4)wk	(7.8 ± 2.4)mo
LVEDd (mm)	44.18 ± 2.50	45.28 ± 2.83	46.88 ± 3.40	48.84 ± 3.26*^†^	46.31 ± 3.14
LVEDs (mm)	28.08 ± 2.84	28.24 ± 3.21	29.62 ± 2.91	30.46 ± 2.81*	28.72 ± 2.51
RWT	0.25 ± 0.09	0.27 ± 0.07	0.28 ± 0.05	0.29 ± 0.06*	0.26 ± 0.06
EF (%)	67.57 ± 5.12	68.00 ± 5.21	65.46 ± 4.49†	64.98 ± 3.93*^†^	66.89 ± 4.91
s (cm/min)	7.0 ± 1.6	7.0 ± 1.2	6.8 ± 1.0	6.4 ± 1.2	6.9 ± 1.7

Data are given as mean ± SD; LVEDd indicates left ventricular end-diastolic dimension; LVEDs, left ventricular end-systolic dimension; RWT, relative wall thickness; EF, ejection fraction; s, peak myocardial velocity.

*P < 0.05 vs. Controls; ^†^ *P* < 0.05 vs. Trimester 1; ^‡^ *P* < 0.05 vs. Trimester 3; ^§^ *P* < 0.05 vs. Postpartum.

### Evolution of 3D echocardiographic assessment

**Figure 3:**
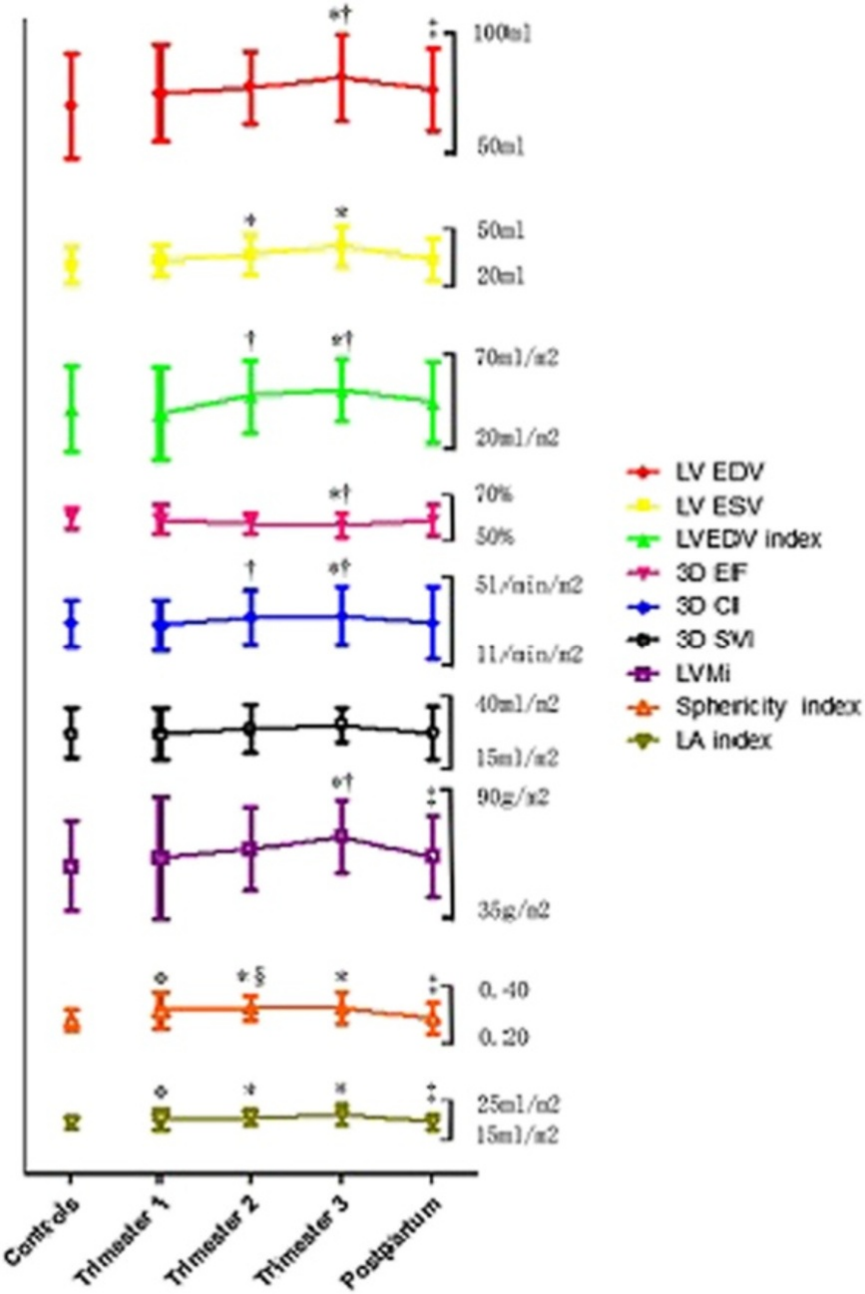
**Evolution of maternal LV morphology and function during pregnancy and postpartum by 3D STE.** Data are presented as mean ± SD. LVEDV indicates left ventricular end-diastolic volume; LVESV, left ventricular end-systolic volume; LVEDVi, left ventricular end-diastolic volume index; EF, ejection fraction; CI, cardiac index; SVI, stroke volume index; LVMi, left ventricular mass index; LA index, left atrial volume index; *P < 0.05 vs. Controls; †*P* < 0.05 vs. Trimester 1; ‡*P* < 0.05 vs. Trimester 3; §*P* < 0.05 vs. Postpartum.

**Figure 4:**
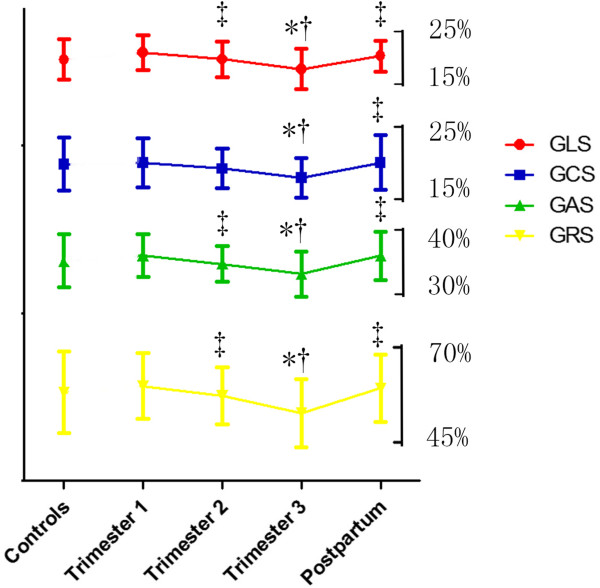
**Assessment of myocardial deformation during pregnancy and postpartum by 3D STE.** Data are presented as mean ± SD. Note that GLS, GCS, GRS and GAS decreased in Trimester 3, while returned in postpartum. GLS indicates global longitudinal strain; GCS, global circumferential strain; GAS, global area strain; GRS, global radial strain. *P < 0.05 vs. Controls; †*P* < 0.05 vs. Trimester 1; ‡*P* < 0.05 vs. Trimester 3; §*P* < 0.05 vs. Postpartum.

3D echocardiography assessment of LV structure and function is reported in Table 3. Indices of LV volume, LV mass index (LVMi), SpI and cardiac index (CI) increased during pregnancy, while 3D EF tended to decrease in the third trimester and consequently recovered postpartum (Figure 3). GLS, GCS, GRS and GAS showed a significant decrease in the second and third trimester, and increased again postpartum (Figure 4). This behavior was found in almost all ventricular walls. The biggest differences were found in the anterior and some segments of inferoseptal, and anteroseptal walls (Table 4). Table 5 summarizes the univariate relations of 3D-derived strain in the pooled population. Among the strain components, GAS showed the strongest associations with 3D EF, SpI and LVMi. GAS showed strong correlations with GLS (*r* = 0.81, *P* < 0.01) and GCS (*r* = 0.82, *P* < 0.01).

**Table 3:** **Real-time three-dimensional echocardiographic assessment**

Variable	Controls	Trimester 1	Trimester 2	Trimester 3	Postpartum
	-	(14.1 ± 1.6)wk	(25.5 ± 2.6)wk	(37.8 ± 2.4)wk	(7.8 ± 2.4)mo
LV EDV(ml)	76.14 ± 21.20	81.39 ± 19.54	83.78 ± 14.58	87.72 ± 17.18*^†^	82.77 ± 16.85^‡^
LV ESV(ml)	32.00 ± 7.29	33.91 ± 6.16	36.30 ± 7.96*	39.67 ± 7.98*	34.08 ± 8.32
LVEDVi(ml/m^2^)	44.06 ± 17.44	41.93 ± 18.61	49.33 ± 14.49^†^	51.67 ± 12.65*^†^	46.63 ± 16.49
3D EF(%)	60.14 ± 4.16	59.48 ± 5.77	58.00 ± 4.06	57.07 ± 4.62*^†^	59.16 ± 6.12
3D CI(l · min^−1^ · m^−2^)	2.26 ± 0.93	2.17 ± 1.02	2.49 ± 1.09^†^	2.54 ± 1.17*^†^	2.27 ± 1.43
3D SVI(ml/m^2^)	27.79 ± 9.96	27.51 ± 10.36	29.58 ± 9.62	31.37 ± 1.73	28.08 ± 11.02
LVMi (g/m^2^)	59.25 ± 18.00	62.57 ± 24.81	66.37 ± 16.74	71.23 ± 14.46*^†^	63.14 ± 16.08^‡^
Sphericity index	0.27 ± 0.04	0.31 ± 0.07*	0.32 ± 0.05*^§^	0.32 ± 0.06*	0.28 ± 0.06^‡^
LA index (ml/m^2^)	11.0 ± 2.5	12.3 ± 4.2	13.1 ± 3.3*^§^	14.2 ± 4.3*^†^	11.2 ± 3.3^‡^
GLS (%)	20.29 ± 2.97	21.32 ± 2.61	20.30 ± 2.66^‡^	18.85 ± 2.99*^†^	20.82 ± 2.31^‡^
GCS (%)	18.71 ± 3.93	18.91 ± 3.70	18.05 ± 2.99	16.65 ± 2.99*^†^	18.91 ± 4.07^‡^
GAS (%)	33.71 ± 5.28	34.77 ± 4.21	33.09 ± 3.56^‡^	31.10 ± 4.53*^†^	34.68 ± 4.81^‡^
GRS (%)	56.36 ± 12.15	58.32 ± 9.88	55.36 ± 8.47^‡^	50.23 ± 10.12*^†^	57.62 ± 10.10^‡^

Data are given as mean ± SD, LVEDV indicates left ventricular end-diastolic volume; LVESV, left ventricular end-systolic volume; LVEDVi, left ventricular end-diastolic volume index; EF, ejection fraction; CI, cardiac index; SVI, stroke volume index; LVMi, left ventricular mass index; LA index, left atrial volume index; GLS, global longitudinal strain; GCS, global circumferential strain; GAS, global area strain; GRS, global radial strain.

*P < 0.05 vs. Controls; ^†^ *P* < 0.05 vs. Trimester 1; ^‡^ *P* < 0.05 vs. Trimester 3; ^§^ *P* < 0.05 vs. Postpartum.

**Table 4:** **Three-dimensional strain deformation: Wall-by-wall analysis**

Variable (%)		Controls	Trimester 1	Trimester 2	Trimester 3	Postpartum
		-	(14.1 ± 1.6)wk	(25.5 ± 2.6)wk	(37.8 ± 2.4)wk	(7.8 ± 2.4)mo
Longitu.						
	Sep	17.42 ± 5.60	16.50 ± 3.72	16.31 ± 6.88	14.26 ± 5.40	17.90 ± 3.60
	lat	18.25 ± 9.16	19.03 ± 8.04	18.57 ± 6.88	16.85 ± 6.07	18.75 ± 6.25
	Ant	19.58 ± 7.52	21.91 ± 7.01	18.06 ± 6.80^†^	17.20 ± 5.48*^†^	20.02 ± 6.32^‡^
	Inf	15.85 ± 6.82	18.94 ± 5.78	14.76 ± 6.49	15.24 ± 6.42	16.45 ± 5.73
	Pos	13.29 ± 8.22	17.69 ± 8.28	15.71 ± 7.47	15.43 ± 7.22	14.18 ± 7.56
	A-s	16.18 ± 6.15	18.07 ± 6.34	15.73 ± 7.90	13.93 ± 6.70	16.69 ± 6.21
Circum.						
	Sep	16.42 ± 8.09	19.10 ± 5.07	16.58 ± 5.77	15.12 ± 6.38	16.44 ± 7.19
	lat	15.38 ± 8.07	18.00 ± 8.83	18.61 ± 9.01	18.27 ± 8.12	17.31 ± 7.68
	Ant	27.08 ± 4.73	20.05 ± 5.32*	20.09 ± 8.55*	18.94 ± 7.66*	24.45 ± 8.71^‡^
	Inf	14.00 ± 6.44	16.50 ± 7.45	14.82 ± 6.51	14.82 ± 7.79	14.42 ± 6.56
	Pos	11.86 ± 7.70	16.88 ± 7.72	17.94 ± 5.45	16.70 ± 7.94	14.36 ± 7.55
	A-s	23.00 ± 7.17	20.60 ± 5.50	19.79 ± 6.67	18.17 ± 6.93	21.47 ± 6.35
Area						
	Sep	30.58 ± 7.01	32.14 ± 4.05	28.17 ± 6.68	25.91 ± 8.59*^†^	31.58 ± 6.71^‡^
	lat	27.50 ± 12.64	31.89 ± 11.36	30.25 ± 10.42	31.20 ± 9.13	29.09 ± 11.04
	Ant	39.67 ± 6.15	37.35 ± 10.70	32.41 ± 8.31*^†^	31.00 ± 7.16*^†^	38.77 ± 6.07^‡^
	Inf	26.69 ± 9.60	29.89 ± 9.43	26.21 ± 7.90	26.85 ± 8.24	26.37 ± 8.62
	Pos	21.14 ± 12.01	31.38 ± 9.83	29.09 ± 7.15	27.67 ± 9.80	22.56 ± 10.09
	A-s	34.55 ± 5.97	35.00 ± 6.48	31.21 ± 8.34	27.40 ± 10.75*^†^	34.16 ± 7.97^‡^
Radial						
	Sep	45.50 ± 14.59	48.76 ± 9.38	41.97 ± 16.43	37.21 ± 15.68*^†^	46.53 ± 9.62^‡^
	lat	49.38 ± 22.28	55.00 ± 27.56	54.61 ± 26.49	52.47 ± 21.30	50.28 ± 24.21
	Ant	70.17 ± 17.75	60.62 ± 22.18	52.19 ± 18.58*	48.90 ± 15.16*^†^	68.48 ± 16.68^‡^
	Inf	39.08 ± 20.18	42.89 ± 17.28	38.18 ± 15.17	39.70 ± 15.37	40.38 ± 18.17
	Pos	32.14 ± 20.34	47.94 ± 21.94	43.81 ± 15.20	41.77 ± 18.32	34.14 ± 19.64
	A-s	55.82 ± 14.21	56.27 ± 14.60	48.74 ± 17.65	41.60 ± 18.95*^†^	56.67 ± 15.27^‡^

Data are given as mean ± SD, Longitu. indicates Longitudinal;Circum., Circumferential; sep, inferoseptum; lat, lateral wall; Ant, anterior wall; Inf, inferior wall; Pos, posterior wall; A-s, anteroseptum.

*P < 0.05 vs. Controls; ^†^ *P* < 0.05 vs. Trimester 1; ^‡^ *P* < 0.05 vs. Trimester 3; ^§^ *P* < 0.05 vs. Postpartum.

**Table 5:** **Univariate relations (r coefficient and significance) of three-dimensional-derived strain components in the pooled population**

Variable	GLS( ***P*** -value)	GCS( ***P*** -value)	GAS( ***P*** -value)	GRS( ***P*** -value)
Gestation period(w)	−0.286(<0.01)	−0.268(<0.01)	−0.299(<0.01)	−0.296(<0.01)
LVmi(g/m2)	0.176(>0.05)	0.133(>0.05)	0.220(<0.05)	0.170(>0.05)
Sphericity index	0.264(<0.01)	0.191(>0.05)	0.328(<0.01)	0.316(<0.01)
LA index(ml/m2)	0.253(<0.01)	0.171(>0.05)	0.258(<0.01)	0.269(<0.01)
3D EF(%)	0.436(<0.01)	0.472(<0.01)	0.549(<0.01)	0.541(<0.01)

*Abbreviations* as in Tables 2, 3, and 4. Values of GLS, GCS, GRS and GAS considered as ‘positive’ (sign +) to build the univariate relations in order to homogenize the results of analyses and strengthen their clinical meaning: the higher the values, the better is the strain deformation independent of the plus/minus sign.

### Reproducibility

For 3D STE measurements, intraobserver and interobserver variability were 7. 2% and 8.4% for LV EDV measurements; 6.8% and 7.6% for 3D EF measurements; 9.1% and 9.8% for LVMi measurements; and 8.1% and 8.6% for SpI measurements, respectively. Meanwhile, for deformation parameters, intraobserver and interobserver variability were 8.0% and 8.9% for GLS measurements, 9.0% and 9.6% for GCS measurements, 7.1% and 8.4% for GAS measurements as well as 9.4% and 11.2% for GRS measurements, respectively.

## Discussion

The present study examined LV structure and systolic function in healthy pregnant individuals by 3D STE assessment of LV volumes, mass and cardiac motions. To the best of our knowledge, this is the first study to use this quantitative approach as an alteration to standard echocardiograph on normal pregnancies. Similar to previous studies [8], increased preload indicators, such as ventricular volumes, and decreased afterload parameters, such as pressure, during normal pregnancy were found in our study. The new approach confirms the increase of LV volume and mass detected also by 2DE but allows an additional, reliable evaluation for all components of myocardial deformation change according to gestational age. Those data will provide reference values for allowing the dynamic assessment of the echocardiographic parameters during pregnancy and the early detection of pregnancy-associated disorders with 3D STE.

### 3D STE assessment of left ventricular morphological and functional changes

In current clinical practice, 2DE is the first-choice technique to diagnose changes of LV geometry, and LVEF is a widely used and prognostic important indicator of LV systolic function. However, these parameters are inextricably linked with and influenced by the load and the geometry of heart and, therefore, only reflect the ventricular function indirectly. In this regard, several studies have successfully shown that 3D STE has better accuracy than 2DE in comparison with reference techniques, such as cardiac magnetic resonance imaging [14]. Moreover, 3D method can easily provide additional insights. In our study, the changes of 3D parameters, such as LV EDV index, LVMi and SpI, indicated progressive eccentric hypertrophy as a response to the hemodynamic demands during pregnancy, which were similar to the previous 2D study [4, 8]. All changes returned to baseline level in the postpartum period. It is noteworthy that these results were derived from the 3D approach based on direct volumetric quantification, which does not depend on any geometry assumption of the LV and is relatively operator-independent due to its semi-automatic methods [15].

### 3D STE assessment of left ventricular myocardial deformation

In the present study, the changes of global directional strain with 3D STE, including longitudinal, circumferential and radial, were assessed. Although the values between the first and second trimester showed mild but no significant reduction in strain, these components decreased significantly from the second to third trimester and increased again postpartum. This finding is different from the previous 2D STE study that reported only LV longitudinal systolic deformation showed a significant decrease in late pregnancy while circumferential and radial strain showed no changes during the study [8]. Furthermore, we identified that GLS, GCS, GRS correlated well with gestational period, 3D EF, LA index and SpI. It is known that LV contraction actually involves complex three-dimensional rotation, contraction and shortening. Detection of cardiac motion by 2D STE on a single tomographic plane is impaired by through-plane motion, which may produce discrepancies in GCS measurements, especially at the LV basal level [9]. Present study also showed that there were partial discordant results of the mean strain values between 3DT STE and 2D STE. Compared to the 2D approach, 3D STE operates with a larger amount of volume data, which makes speckles in the myocardium that can be tracked in 3D space, and thereby has an advantage to overcome out-of-plane motion. Moreover, by using the 2D imaging, evaluating GLS, GCS and GRS at multiple levels in different heart beat and the different impact of the undetectable displacement vector lead to less reproducible 2D STE measurements [16]. In contrast, encompassing the entire LV myocardium in full-volume data, 3D STE is not influenced by measurement variability caused by variations in 2D image planes recorded at different time points [17] and allows us to obtain a homogeneous spatial distribution of all myocardial displacement vectors from a single volume data [8].

Another new and important finding of our study is represented by the evidence of varied GAS during normal pregnancy. GAS is a novel 3D STE index which corresponds to the percentage of deformation in LV endocardial surface area. Since it has integrated longitudinal and circumferential deformation, Seo et al. inferred that GAS might decrease the tracking error and emphasize synergistically the magnitude of deformation [18]. Several studies have validated that GAS correlates very well with LVEF, BP and wall motion score index [19, 20], but also has the capability of detecting early and subtle LV systolic dysfunction and greater feasibility than other strain parameters [21]. In the present study, GAS showed a significant decrease in late pregnancy, with a 10.56 percentage reduction of mean GAS accompanied by a just 4.05% decrease in mean 3D EF between the first and third trimester, and then it returned to baseline level after delivery. We further found that, compared to other strain components, GAS exhibited the strongest association with 3D EF and SpI, but also was the only parameter of myocardial deformation correlated well with LVMi.

By using 3D method, GLS, GAS, GRS showed small, but significant decreases between the second and third trimester, while EF showed no significant changes at the same time. These data are in concordance with other studies that strain measurements detect subtle changes more sensitively than EF [8, 22]. All the parameters obtained by 3D STE had good reproducibility and therefore appear sufficiently reliable to be used in early detection of pregnancy-associated disorders, such as peripartum cardiomyopathy.

### Why the changes of myocardial deformation happen during pregnancy

We reasoned that the variation of strain during uncomplicated pregnancy could be due to a combination of mechanisms: preload/afterload of ventricle, wall thickness and chamber size.

To produce the same stroke volume, there is an inverse relationship between heart size and strain [23]. Between the first and second trimester, decreased afterload (down-regulated mean blood pressure) is balanced by mild magnified ventricular size and accelerated heart rate, while SV index does not significantly increase. As a result, no notable variation of myocardial deformation occurs as response to the changes of hemodynamic and chamber sizes.

In contrast, despite an increasing stroke work, the values of 3D STE appeared significantly reduced in the late term accompanied by a slight drop of 3D EF. Hypertrophy is commonly seen as a primary mechanism of the heart to reduce stress on the ventricular walls [24]. From the second to third trimester, cardiac hypertrophy is triggered, leading to slight increment of wall thickness, which, however, could not completely compensate for enlargement of chamber and higher afterload. All components of 3D strain, influenced by the unbalance, therefore exhibited significant decrease during late pregnancy. The inconsistencies in prior studies exploring maternal cardiac function may reflect assessments at different gestation age during the third trimester. In addition, the variation of segmental strain reflected changes of regional loading conditions, not only ventricular preload and afterload, but also chamber size and wall thickness [25].

### Limitations

There are several limitations in this study that should be addressed. Firstly, we did not use a non-echocardiographic method for EF estimation to verify the accuracy of 3D measurements, but 3D echocardiography has been validated against cardiac magnetic nuclear resonance imaging [14]. Secondly, because it will take 6 months up to 1 year [26] for hemodynamic and morphological indices to return to baseline after delivery, some parameters in our study might underestimate postpartum changes. Thirdly, there are two important technical limitations of 3D STE. One is that the speckle-tracking analysis is highly dependent on image quality; another is the low frame rate of 3D STE. Both of them could cause miscorrelation between frames and accuracy of strain data. Forthly, we used vender-specific speckle-tracking software, so our findings may not apply to analysis by other vender-independent software. In addition, the differences are indeed statistically significant, but in terms of absolute values, the differences are small and with large overlap, which somehow limits a strong clinical application. Finally, our study only covered a relatively small number of pregnant women in a single-centre, so a large study in the future is required to confirm our results. The value of 3D STE in different pregnancy-associated pathologies should be studied in the further.

## Conclusions

This study provides reference data concerning physiological changes in LV structure and systolic function during normal pregnancy and presents normal range of maternal cardiac motion with 3D STE. Cardiac hypertrophy in pregnancies was observed. We could show that the subtle reduction of myocardial deformation appears as an adaptive response to changes of preload, afterload and LV geometry, and consequently contribute to the modest decrease of EF in late pregnancy. In future clinical use of 3D STE for the early detection of pregnant complications, normal values would require to be adjusted to the gestational age or considering LV geometry and load.

## Abbreviations

LV: Left ventricular
CO: Cardiac output
EF: Ejection fraction
2DE: Two-dimensional echocardiography
3D: Three-dimension
STE: Speckle-tracking echocardiography
IVSd: Interventricular septum
PWd: Posterior wall
LVEDd: Left ventricular end-diastolic diameter
LVEDs: Left ventricular end-systolic diameter
SV: Stroke volume
RWT: Relative wall thickness
EDV: End-diastolic volume
ESV: End-systolic volume
SpI: Sphericity index
GLS: Global longitudinal strain
GCS: Global circumferential strain
GAS: Global area strain
GRS: Global radial strain
LVMi: Left ventricular mass index
CI: Cardiac index.
